# Diagnosis and Treatment of Fungus and Virus Interaction

**DOI:** 10.3390/jof8060620

**Published:** 2022-06-10

**Authors:** Chih-Cheng Lai, Wen-Liang Yu

**Affiliations:** 1 Department of Internal Medicine, Kaohsiung Veterans General Hospital, Tainan Branch, Tainan 71051, Taiwan; dtmed141@gmail.com; 2 Department of Intensive Care Medicine, Chi Mei Medical Center, Tainan 71004, Taiwan; 3 Department of Medicine, School of Medicine, College of Medicine, Taipei Medical University, Taipei 11031, Taiwan

Many viruses can have a serious impact on human respiratory disease, e.g., Influenza A (H1N1) causing severe pneumonia and SARS-CoV-2 causing COVID-19 [1,2]. In addition to these respiratory viruses’ direct associated damage, the virus–host interplay may make the patient vulnerable to further develop a co- or secondary fungal infection. This highlights the importance of updating the current knowledge on the epidemiology and outcome of post-viral fungal infections. This present Special Issue of the *Journal of Fungi* addresses the clinical interaction between viruses and fungi. Studies cover new epidemiological knowledge, diagnosis, and treatment of virus-associated fungal diseases, including fungal diseases with COVID-19 in four articles [3,4,5,6] and with Influenza A (H1N1) in two studies [7,8].

First, Baghel et al. report the clinical syndrome of COVID-19-associated invasive fungal sinusitis from patients at a tertiary care center in northern India [3]. Between April 2021 and September 2021, a total of 124 patients who were either COVID-19-positive or had recovered from COVID-19 and developed invasive fungal sinusitis were included [3]. Among them, 83.9% had diabetes mellitus, and 72.6% had previously received steroids. Uncontrolled glycemic levels and steroid use were the most important predisposing factors of COVID-19 co- or secondary invasive fungal sinusitis. Mucormycosis was the most common causative fungal disease, which accounts for 92%, followed by aspergillosis (16.9%) and hyalohyphomycosis (0.8%). In addition, 16 (12.9%) patients had both mucormycosis and aspergillus. Maxillary sinus was the most common site involved (*n* = 90, 72.6%), followed by ethmoid sinuses 87 (70.2%). The overall three-month survival rate was 79.9%. These findings remind clinicians to keep alert for the occurrence of invasive fungal sinusitis in patients with COVID-19 or even recovered COVID-19, particularly for those with poor glycemic control or receiving steroids [3].

Second, Marta et al. reported another important issue—COVID-19-associated pulmonary aspergillosis (CAPA) [4]. This prospective study was conducted at a tertiary university hospital in Spain during the first and second wave of COVID-19 and included a total of 300 patients with COVID-19 who had acute respiratory failure and required intensive care unit (ICU) admission. Among them, 35 patients were diagnosed with CAPA, and the overall prevalence was 11.7%. In multivariate analysis, the independent risk factors for CAPA included age (OR: 1.05; 95% CI 1.01–1.09; *p* = 0.037), chronic lung disease (OR: 3.85; 95% CI 1.02–14.9; *p* = 0.049) and treatment with tocilizumab during admission (OR: 14.5; 95% 6.1–34.9; *p* = 0.001). Mycological culture was positive in 34 patients (97.1%), in which 27 (80%) had grew one *Aspergillus*, and 7 patients (20%) had more than one species of *Aspergillus*. *Aspergillus fumigatus* was the most common pathogen, followed by *Aspergillus terreus* and *Aspergillus niger* and *Aspergillus nidulans.* The mortality rate of CAPA was 31.4%, which was significantly associated with age (OR: 1.06; 95% CI 1.01–1.11; *p* = 0.014) and CAPA diagnosis on admission (OR: 3.34; 95% CI 1.38–8.08; *p* = 0.007). These findings indicated that CAPA was not uncommon among patients with critical COVID-19 in the ICUs, and the mortality of CAPA remained high. Similar findings were shown in the two other series in Taiwan [5,6]. A single-center, retrospective study by Wu et al. reported that 6 (20.7%) of 29 mechanically ventilated COVID-19 patients with severe pneumonia had coinfection with CAPA based on elevated serum galactomannan levels and/or bronchoalveolar lavage fluid [5]. In this study, the relatively high prevalence of CAPA is probably due to aggressively regular check-ups for *Aspergillus* galactomannan antigen [5]. However, the authors did not find conclusive evidence about the association between tocilizumab and the risk of CAPA. Another retrospective study by Lu et al. reviewed 36 mortality cases of 178 hospitalized COVID-19 patients in a medical center between January 2020 and September 2021 [6]. Among them, only one *Aspergillus* (2.7%) species was identified in the respiratory pathogens. Overall, the above findings should suggest the importance of screening aspergillosis for severe or critical COVID-19 patients. However, the prevalence of CAPA might be underestimated because the diagnosis of CAPA was made only based on cultural methods.

In addition to SARS-CoV-2, the association between severe influenza and invasive pulmonary aspergillosis should be seriously concerned. Previous studies have revealed higher mortality rates in patients with influenza-associated pulmonary aspergillosis (IAPA) than in patients with severe influenza alone [9]. In this Special Issue, there were two articles on IAPA reported from two medical centers in Taiwan [7,8]. First, Wu et al. reported 24 (11.2%) culture-positive IAPA patients out of 215 patients with severe influenza from 2016 to 2019, mostly infected by *A. fumigatus* (62.5%), *A. flavus* (25.0%), and *A. terreus* (16.7%). The mean time from influenza diagnosis to *Aspergillus* growth was 4.4 days. Pure *Aspergillus* growth without bacterial co-isolation in culture was found in 17 (70.8%) patients. Although all patients received voriconazole, the all-cause mortality was up to 41.6%. This finding suggested that IAPA is an early and rapidly deteriorating complication following influenza that necessitates prompt workup [8]. Second, Chao et al. reported the impacts of IAPA on economic burden and risk factors for mortality in critically ill patients from 2016 to 2018 [7]. The risk factors for IAPA were solid cancer and prolonged corticosteroid use. IAPA patients had a significantly higher mortality rate (20/40, 50%) than influenza patients without IAPA (6/50, 12%). IAPA patients had significantly longer hospital stays and higher economic burdens than the controls. The risk factors for mortality in IAPA patients included the Charlson index, APACHE II score, and severe acute respiratory distress syndrome. Therefore, severe influenza patients should be promptly managed based on risk factors for the occurrence of IAPA and its mortality.

In summary, this Special Issue highlights important work regarding invasive fungal diseases following infections with influenza or SARS-CoV-2. We highly appreciate all authors and reviewers for their significant contributions to this Special Issue. Hopefully, this Special Issue is a valuable collection of studies that are interesting to read and to learn from.

## Data Availability

Not applicable.
